# Omega-3 fatty acids as modulators of advanced glycation end products in aging: mechanistic pathways and clinical implications - a narrative review

**DOI:** 10.3389/fragi.2026.1872170

**Published:** 2026-07-14

**Authors:** Anna Cortesi, Irene P. Tzanetakou, Konstantinos Giannakou, Aikaterini Bograkou-Tzanetakou, Elena Hadjimbei

**Affiliations:** 1 Laboratory of Experimental Surgery and Surgical Research “N.S. Christeas”, Medical School, National and Kapodistrian University of Athens, Athens, Greece; 2 Department of Life Sciences, School of Sciences, European University Cyprus, Nicosia, Cyprus; 3 Department of Health Sciences, School of Sciences, European University Cyprus, Nicosia, Cyprus; 4 School of Medicine, European University Cyprus, Nicosia, Cyprus

**Keywords:** AGEs receptor (RAGE), advanced glycation end products receptor (RAGE), aging, elderly, inflammation, omega-3 polyunsaturated fatty acids (PUFAs), oxidative stress

## Abstract

**Background:**

Aging is characterised by the progressive accumulation of advanced glycation end products (AGEs), formed through non-enzymatic glycation reactions between reducing sugars and proteins, lipids, or nucleic acids. AGEs contribute to tissue damage through irreversible protein cross-linking and receptor-mediated inflammatory signalling via the receptor for AGEs (RAGE). Elderly individuals are disproportionately affected due to cumulative oxidative stress, chronic low-grade inflammation (inflammaging), impaired renal and enzymatic clearance, and prolonged exposure to dietary AGEs. Omega-3 polyunsaturated fatty acids (PUFAs), particularly eicosapentaenoic acid (EPA) and docosahexaenoic acid (DHA), may modulate these pathways through anti-inflammatory, antioxidant, and metabolic mechanisms.

**Objectives:**

To synthesise available preclinical, *in vitro*, and clinical evidence on the mechanistic and therapeutic relationships between omega-3 PUFA supplementation and AGE formation, accumulation, and signalling, and to evaluate the effects of omega-3 supplementation on key age-related clinical outcomes in elderly populations, including cardiometabolic health, cognitive function, mental health, functional capacity, and quality of life.

**Methods:**

A narrative review was conducted using searches of PubMed, Scopus, and Web of Science. Eligible study designs included *in vitro* experiments, animal models, randomised controlled trials (RCTs), observational studies, and systematic reviews published in English. Evidence was synthesised thematically across mechanistic and clinical domains, with priority given to studies addressing AGE-related pathways and clinically relevant endpoints.

**Results:**

Preclinical evidence demonstrates that omega-3 PUFAs attenuate oxidative stress and inflammatory signalling, partly through RAGE axis regulation and PPARγ activation. Clinical studies report reductions in circulating AGEs, RAGE, and pentosidine following supplementation, alongside upregulation of protective scavenger receptors such as AGER1. Omega-3 supplementation has also been associated with improvements in inflammatory markers, cardiometabolic risk, cognitive function, and physical capacity in older adults. However, RCT evidence assessing tissue-level AGE accumulation using non-invasive measures such as skin autofluorescence remains limited in elderly populations.

**Conclusion:**

Omega-3 PUFAs demonstrate therapeutic potential in modulating AGE-related mechanisms and improving age-associated outcomes. Well-designed RCTs targeting tissue AGE accumulation and long-term endpoints in elderly cohorts are warranted.

## 1 Introduction

The global population is aging at an unprecedented rate. According to the World Health Organization, the proportion of individuals aged 60+ years reaching 22% by 2050, with particularly steep increases in the population residing in long-term care facilities (WHO, 2022). This demographic transformation is accompanied by a profound increase in the burden of age-related chronic diseases, including cardiovascular disease, type 2 diabetes, cognitive decline, sarcopenia, and frailty, imposing significant challenges on healthcare systems worldwide (Lopez-Otin et al., 2023).

Aging is a complex, multifactorial biological process characterized by the gradual accumulation of cellular damage, impaired proteostasis, mitochondrial dysfunction, chronic low-grade systemic inflammation termed inflammaging, and increased oxidative stress (Franceschi et al., 2018; Lopez-Otin et al., 2013). These interconnected mechanisms converge to promote the formation and accumulation of advanced glycation end products (AGEs), a chemically heterogeneous class of compounds arising from non-enzymatic reactions between reducing sugars and macromolecules such as proteins, lipids, and nucleic acids (Singh et al., 2001; Vlassara and Uribarri, 2014). The accumulation of AGEs in tissues is not merely a marker of metabolic dysfunction but an active driver of disease, mediating pathological effects through direct structural cross-linking of long-lived proteins and through receptor-mediated signalling via the receptor for AGEs (RAGE), which perpetuates oxidative stress and inflammation (Ramasamy et al., 2005).

Omega-3 polyunsaturated fatty acids (PUFAs), principally eicosapentaenoic acid (EPA) and docosahexaenoic acid (DHA), have long been recognized as key regulators of inflammatory and oxidative pathways. By incorporating into cell membrane phospholipids, they alter membrane fluidity and receptor signalling, serve as precursors to specialized pro-resolving lipid mediators (SPMs), and suppress NF-κB-mediated transcription of pro-inflammatory cytokines (Calder, 2017). Their potential to modulate AGE formation and accumulation, while simultaneously improving cardiometabolic, cognitive, and functional health outcomes in the elderly, represents a compelling but insufficiently investigated research area.

The present narrative review synthesizes existing evidence on: (1) the biology of AGEs and their measurement, with particular focus on skin autofluorescence as a non-invasive clinical tool; (2) the role of omega-3 fatty acids in aging, inflammation, and oxidative stress; (3) the direct evidence for omega-3 modulation of AGE accumulation; and (4) the effects of omega-3 supplementation on key secondary outcomes including cardiometabolic health, cognitive function, functional capacity, and quality of life in elderly populations.

## 2 Methods

This article is a narrative review informed by a structured literature search. The objective was not to systematically evaluate the effectiveness of a single intervention on a narrowly defined outcome, but rather to provide an integrative synthesis of mechanistic, translational, and clinical evidence regarding the relationship between omega-3 fatty acids, advanced glycation end products (AGEs), and healthy aging. The topic encompasses heterogeneous sources of evidence, including molecular studies, *in vitro* experiments, animal models, clinical trials, observational studies, and existing systematic reviews, with considerable variation in populations, formulations, dose ranges, and endpoints. Given this breadth and heterogeneity, a narrative review methodology was considered more appropriate than a systematic or scoping review, as it allowed the integration of mechanistic pathways with clinical implications and the development of a comprehensive conceptual framework. A brief methods description is retained for transparency, to describe the databases searched, the terms used, and the prioritisation criteria applied, consistent with best practice in narrative reviews (Baethge et al., 2019).

A literature search was conducted in PubMed, Scopus, and Web of Science from database inception up to January 2026, using combinations of the following terms: “omega-3”, “EPA”, “DHA”, “n-3 PUFA”, “advanced glycation end products”, “AGEs”, “RAGE”, “skin autofluorescence”, “aging”, “oxidative stress”, and “inflammation”.

Studies were prioritised based on the following criteria:

- Direct assessment of AGE formation, accumulation, or AGE–RAGE signalling
- Evaluation of omega-3 PUFA interventions (EPA, DHA)
- Reporting of AGE-related biomarkers (e.g., CML, pentosidine, RAGE, skin autofluorescence)

Eligible study designs included *in vitro* studies, animal models, randomized controlled trials (RCTs), and clinical trials. PRISMA-style exhaustive reporting was not undertaken, consistent with the narrative review design.

## 3 Aging, inflammaging, and oxidative stress

### 3.1 The biology of aging

Aging is recognized as a complex, progressive deterioration of physiological function driven by the interaction of intrinsic genetic programs and extrinsic environmental stressors. Lopez-Otin et al. (2013) identified nine hallmarks of aging including genomic instability, telomere attrition, epigenetic alterations, loss of proteostasis, deregulated nutrient sensing, mitochondrial dysfunction, cellular senescence, stem cell exhaustion, and altered intercellular communication, which together delineate the cellular and molecular landscape of the aging process. A subsequent update expanded these hallmarks to include disabled macroautophagy, chronic inflammation, and dysbiosis (Lopez-Otin et al., 2023), reflecting the growing appreciation of systemic factors in biological aging.

A central consequence of aging is the accumulation of oxidative damage. Oxidative stress arises when the production of reactive oxygen species (ROS) and reactive nitrogen species exceeds the capacity of endogenous antioxidant defense systems, including superoxide dismutase (SOD), catalase, glutathione peroxidase (GPx), and non-enzymatic antioxidants such as vitamins C and E (Sies et al., 2017). The mitochondrial electron transport chain is the primary cellular source of ROS production; with aging, mitochondrial efficiency declines and electron leakage increases, amplifying the oxidative burden on cells and tissues (Bratic and Larsson, 2013). This state of chronic, low-level oxidative stress is a prerequisite for the accelerated formation of AGEs.

### 3.2 Inflammaging: chronic low-grade inflammation and aging

In 2000, Franceschi and colleagues introduced the concept of inflammaging to describe the phenomenon of sterile, chronic, low-grade systemic inflammation that accompanies aging and underlies the pathogenesis of most age-related diseases (Franceschi et al., 2000). Inflammaging is characterized by elevated circulating concentrations of pro-inflammatory cytokines including interleukin-6 (IL-6), tumor necrosis factor-alpha (TNF-α), interleukin-1 beta (IL-1β), and C-reactive protein (CRP), in the absence of overt infection or acute injury (Ferrucci and Fabbri, 2018).

The molecular mechanisms driving inflammaging are diverse and interconnected. They include the accumulation of senescent cells that secrete the senescence-associated secretory phenotype (SASP), impaired clearance of cellular debris and misfolded proteins due to reduced autophagic flux, activation of the NLRP3 inflammasome by endogenous danger signals (DAMPs), mitochondrial dysfunction-induced cytokine production, gut microbiome dysbiosis increasing intestinal permeability and systemic endotoxin exposure, and increased central adiposity amplifying adipokine-driven inflammatory signalling (Franceschi et al., 2018; Ferrucci and Fabbri, 2018). Critically, inflammation and oxidative stress are mutually reinforcing: NF-κB activation by ROS drives transcription of pro-inflammatory mediators, while inflammatory cytokines stimulate further ROS production through activation of NADPH oxidase and mitochondrial uncoupling.

In older adults, inflammaging is further exacerbated by physical inactivity, nutritional deficiencies, polypharmacy, social isolation, and the high burden of chronic comorbidities (Bauer et al., 2022). These factors collectively accelerate AGE formation and tissue accumulation, making this population a particularly relevant target for dietary interventions aimed at reducing glyco-oxidative stress.

## 4 Advanced glycation end products (AGEs)

### 4.1 Formation and classification

Advanced glycation end products are a structurally diverse family of compounds formed through the Maillard reaction, a series of non-enzymatic condensation and oxidation reactions between the carbonyl groups of reducing sugars (primarily glucose, fructose, and ribose) and the free amino groups of proteins, lipids, or nucleic acids (Brownlee, 1995; Singh et al., 2001). The reaction proceeds through a sequence of intermediate Amadori products and Schiff base formation, ultimately yielding a heterogeneous mixture of AGE compounds including N-(carboxymethyl)lysine (CML), N-(carboxyethyl)lysine (CEL), pentosidine, glyoxal-lysine dimer (GOLD), and methylglyoxal-lysine dimer (MOLD), among many others (Nowotny et al., 2018).

AGEs are broadly classified as fluorescent and non-fluorescent. Fluorescent AGEs, such as pentosidine and crossline, exhibit characteristic excitation and emission spectra that form the basis for non-invasive measurement technologies (Meerwaldt et al., 2004). Non-fluorescent AGEs, such as CML, are the most abundant AGEs in human tissue and are commonly quantified using immunoassays. Both endogenous AGE formation driven by hyperglycemia, oxidative stress, and aging, and exogenous AGE ingestion through the diet contribute to the total body AGE burden (Uribarri et al., 2010).

Diet represents a significant exogenous source of AGEs. Foods processed at high temperatures through frying, grilling, roasting, or baking undergo extensive Maillard browning, generating abundant dietary AGEs (Goldberg et al., 2004). Approximately 10% of ingested AGEs are absorbed from the gastrointestinal tract and enter the circulation, where they may accumulate in tissues or be excreted via the kidneys (Uribarri et al., 2010). Reduced renal function, common in older adults, impairs AGE clearance and amplifies tissue accumulation. Conversely, low-temperature cooking methods such as boiling or steaming substantially reduce dietary AGE content, and a Mediterranean dietary pattern, rich in antioxidants and low in high-temperature processed foods, is associated with significantly lower circulating and tissue AGE levels (Poulsen et al., 2013).

### 4.2 Pathophysiological mechanisms of AGEs

The deleterious effects of AGE accumulation operate through two principal mechanisms: (1) direct modification of structural proteins through cross-linking, and (2) receptor-mediated cellular activation via RAGE. Cross-linking of long-lived proteins in the extracellular matrix, particularly collagen and elastin, impairs their normal structural and functional properties, increasing tissue stiffness, reducing elasticity, and disrupting cell-matrix interactions (Vlassara and Uribarri, 2014). The consequences are particularly pronounced in connective tissues of the vasculature, skin, cartilage, and lens, contributing to arterial stiffness, skin fragility, sarcopenia, and cataract formation, respectively (Bailey et al., 1998; Ramasamy et al., 2005).

RAGE is a multiligand receptor belonging to the immunoglobulin superfamily, expressed on diverse cell types including endothelial cells, smooth muscle cells, cardiomyocytes, neurons, and immune cells. Upon binding of AGEs, as well as other DAMPs including HMGB1 and S100 proteins, RAGE activates multiple downstream signalling cascades including NF-κB, MAPK, and JAK-STAT pathways, resulting in sustained transcription of pro-inflammatory cytokines, adhesion molecules (VCAM-1, ICAM-1), and pro-oxidant enzymes (Ramasamy et al., 2005; Schmidt et al., 1999). This creates a self-perpetuating cycle in which AGE-RAGE signalling drives further oxidative stress and AGE formation, amplifying tissue damage over time.

The pathophysiological consequences of chronic AGE-RAGE axis activation span virtually all organ systems. In the vasculature, AGEs promote endothelial dysfunction, foam cell formation, and plaque destabilization, accelerating atherosclerosis (Ramasamy et al., 2005). In the kidney, mesangial expansion and glomerular basement membrane thickening drive the progression of diabetic nephropathy (Schmidt et al., 1999). In the nervous system, AGEs have been detected in amyloid plaques and neurofibrillary tangles in Alzheimer’s disease brains, and RAGE-mediated neuroinflammation is implicated in neurodegeneration (Li et al., 2012; Mooldijk et al., 2024). In skeletal muscle, collagen AGE cross-linking impairs muscle fibre mechanics and regeneration, contributing to sarcopenia (Bailey et al., 1998).

In addition to the pro-inflammatory RAGE signalling pathway, a counter-regulatory AGE receptor system plays an important role in AGE clearance and detoxification. AGER1 (AGE receptor 1) functions as a scavenger receptor that promotes AGE uptake and intracellular degradation while suppressing intracellular oxidative signalling, thereby limiting the perpetuation of the AGE-RAGE inflammatory cycle. Soluble RAGE (sRAGE), a decoy isoform generated by proteolytic cleavage or alternative splicing, may competitively bind circulating AGE ligands and HMGB1 before they engage membrane-bound RAGE, thus attenuating downstream pro-inflammatory signalling. The ratio of signalling RAGE to protective AGER1 and sRAGE represents a biologically meaningful balance that shifts towards a pro-inflammatory state in aging and metabolic disease, and that may be amenable to dietary modulation. As discussed in Section 6.2, omega-3 supplementation has been associated with upregulation of AGER1 and changes in sRAGE levels, providing mechanistic context for its AGE-modulating effects.

### 4.3 Methods for the assessment of advanced glycation end products: From invasive to non-invasive approaches

A range of methodologies has been developed for the assessment of AGEs, reflecting their structural heterogeneity and diverse biological distribution. These approaches can be broadly categorized into tissue-based measurements, circulating biomarkers, analytical quantification techniques, and non-invasive methods, each providing complementary insights into AGE burden.

Measurement of tissue-bound AGEs, particularly in long-lived proteins such as dermal collagen, is considered the reference standard for assessing cumulative glycation. This is typically performed via skin biopsy followed by biochemical or fluorescence-based analysis of AGE modifications, including pentosidine, Nε-(carboxymethyl)lysine (CML), and crosslinking structures (Monnier et al., 1999; Sell and Monnier, 2012). As dermal collagen has a slow turnover rate, tissue AGE levels reflect long-term glyco-oxidative exposure. However, despite its analytical accuracy, this approach is invasive, technically demanding, and not suitable for routine clinical use or large-scale studies.

Circulating AGEs, measured in plasma or serum, provide a more accessible alternative and are widely used in clinical and epidemiological research. Commonly assessed markers include CML, Nε-(carboxyethyl)lysine (CEL), and pentosidine, which can be quantified using enzyme-linked immunosorbent assays (ELISA), high-performance liquid chromatography (HPLC), or liquid chromatography–mass spectrometry (LC–MS/MS) (Henle, 2003; Nowotny et al., 2018). Among these, LC–MS/MS offers superior specificity and sensitivity. Nevertheless, circulating AGE concentrations are influenced by short-term metabolic fluctuations, renal clearance, and dietary intake, and may therefore not accurately reflect long-term tissue accumulation.

Analytical techniques targeting AGE fluorescence have also been developed, exploiting the intrinsic fluorescent properties of certain AGE compounds. These methods provide an indirect estimate of AGE burden but are limited by their inability to detect non-fluorescent AGEs, which constitute a substantial proportion of total AGE content.

In response to these limitations, non-invasive approaches have gained increasing attention, particularly in the context of aging and chronic disease research. Among these, skin autofluorescence (SAF) has emerged as a validated and clinically applicable method for estimating tissue AGE accumulation *in vivo*. SAF measures the fluorescent emission of AGEs accumulated in dermal collagen and has been shown to correlate strongly with biopsy-derived AGE levels (Meerwaldt et al., 2004; Koetsier et al., 2010). Given its non-invasive nature, rapid assessment, and reproducibility, SAF is increasingly used in both clinical and population-based studies, particularly in older adults.

While SAF does not capture non-fluorescent AGE species, it provides a practical surrogate marker of cumulative glyco-oxidative stress. In this context, non-invasive techniques such as SAF represent an attractive and clinically feasible alternative to invasive tissue-based methods, supporting their growing role in translational and geroscience research. However, an important measurement validity concern is that melanin-related optical interference at SAF excitation wavelengths may affect the accuracy and reliability of SAF measurements in individuals with darker skin tones, resulting in greater measurement variability and potential bias in the estimation of skin AGE accumulation. Although correction algorithms have been developed to account for skin pigmentation, residual measurement error remains, particularly in individuals with highly pigmented skin types (Atzeni et al., 2020). This limitation is particularly relevant when designing trials in ethnically diverse elderly populations. Future studies should consider stratification by Fitzpatrick skin phototype, inclusion of complementary circulating AGE-related biomarkers such as CML and sRAGE alongside SAF measurements, and methodological refinements to improve the validity of non-invasive AGE assessment across different skin types.

Importantly, the choice of AGE assessment method has direct implications for the interpretation of both mechanistic and clinical evidence. While circulating AGE markers primarily reflect short-term metabolic status, tissue-based and non-invasive measures such as SAF provide a more integrated estimate of cumulative glyco-oxidative burden. This distinction is particularly relevant when evaluating interventions such as omega-3 fatty acid supplementation, which may exert both acute metabolic effects and longer-term structural modifications. As discussed in the following sections, understanding how omega-3 PUFAs influence AGE formation, accumulation, and AGE–RAGE signalling pathways requires careful consideration of the methodological context in which AGE-related outcomes are assessed.

## 5 OMEGA-3 fatty acids: Biochemistry and mechanisms of action

### 5.1 Sources, metabolism, and bioavailability

Omega-3 PUFAs are characterized by the presence of the first double bond at the third carbon from the methyl terminus of the fatty acid chain. The principal dietary omega-3 fatty acids relevant to human health are alpha-linolenic acid (ALA, 18:3n-3), a short-chain precursor found in plant foods including flaxseed, walnuts, and canola oil; and the long-chain PUFAs EPA (20:5n-3) and DHA (22:6n-3), found predominantly in fatty fish, fish oils, krill oil, and certain algal sources (Calder, 2017). Humans possess limited capacity to convert ALA to EPA and DHA via elongation and desaturation enzymes, with conversion efficiencies estimated at 0.3%–8% for EPA and below 0.1% for DHA, making direct consumption of long-chain omega-3s essential for therapeutic purposes (Burdge and Calder, 2005).

Upon ingestion, EPA and DHA are absorbed and incorporated into plasma phospholipids, lipoproteins, and red blood cell membranes within hours. With sustained supplementation, they progressively replace arachidonic acid (ARA, 20:4n-6) and other n-6 PUFAs in tissue phospholipid pools, a process requiring weeks to months to reach steady state (Calder, 2017). The omega-3 index, the percentage of EPA + DHA in erythrocyte membrane fatty acids, is the preferred biomarker of omega-3 status, with an index above 8% associated with cardioprotective effects (Harris and von Schacky, 2004). In elderly individuals, omega-3 bioavailability may be affected by impaired gastrointestinal absorption, altered lipid metabolism, and reduced compliance with supplementation regimens.

Formulation type substantially influences bioavailability and should be considered when interpreting trial evidence and designing future interventions. Ethyl ester (EE) formulations, which are the most commonly studied in clinical trials, exhibit lower bioavailability compared with natural triglyceride (TG) forms under fasting conditions, with absorption approximately 25%–73% inferior to TG forms in the absence of co-ingested dietary fat. Phospholipid-bound preparations, such as those derived from krill oil, may offer distinct tissue distribution profiles and potentially superior central nervous system bioavailability due to structural similarity with brain membrane phospholipids. Prescription-grade pure EPA formulations (e.g., icosapentaenoic acid ethyl ester) differ pharmacologically from mixed EPA + DHA over-the-counter products and have demonstrated independent cardiovascular benefit in large RCTs. These distinctions may be especially relevant for therapeutic efficacy in elderly individuals with impaired fat absorption, reduced gastric acid secretion, and altered bile acid metabolism, and should be explicitly reported in future trials.

### 5.2 Anti-inflammatory mechanisms

The anti-inflammatory properties of EPA and DHA are mediated through multiple, partially overlapping mechanisms. At the membrane level, their incorporation into phospholipids reduces the availability of ARA for conversion by cyclooxygenase (COX) and lipoxygenase (LOX) enzymes into pro-inflammatory eicosanoids, including prostaglandins, thromboxanes, and leukotrienes of the 2-series and 4-series (Calder, 2013). EPA and DHA are themselves substrates for COX-3 and 5-LOX, generating eicosanoids of the 3-series and 5-series with substantially weaker inflammatory potency.

Beyond eicosanoid competition, EPA and DHA are precursors for a family of bioactive lipid mediators, including resolvins (E and D series), protectins, and maresins, collectively termed specialized pro-resolving mediators (SPMs), which actively resolve inflammation and promote tissue homeostasis (Serhan, 2017). DHA-derived neuroprotectin D1 (NPD1) is particularly relevant to neurological health, promoting neuronal survival and inhibiting apoptotic signalling in the aging brain.

At the transcriptional level, EPA and DHA inhibit NF-κB activation, the master regulator of pro-inflammatory gene expression, through multiple upstream targets including Toll-like receptor 4 (TLR4) signalling, inhibition of IκB kinase, and activation of the nuclear receptor PPARγ, which suppresses inflammatory gene transcription (Calder, 2013; Xue et al., 2012). They also upregulate AMPK/SIRT1 signalling pathways, which have anti-inflammatory and anti-aging properties. The net effect is a reduction in transcription of pro-inflammatory cytokines (IL-6, TNF-α, IL-1β), adhesion molecules, and chemokines, corresponding to measurable reductions in circulating CRP, IL-6, and TNF-α observed in clinical trials (Calder et al., 2020).

### 5.3 Antioxidant properties and relevance to AGE formation

The antioxidant properties of EPA and DHA are increasingly recognized as a key dimension of their health effects, with direct mechanistic relevance to AGE formation. Omega-3 PUFAs activate the Nrf2 (nuclear factor erythroid 2-related factor 2) pathway, the master transcriptional regulator of cellular antioxidant defense, inducing the expression of antioxidant enzymes including heme oxygenase-1 (HO-1), NAD(P)H quinone dehydrogenase 1 (NQO1), SOD, catalase, and GPx (Chen et al., 2020). By reducing the cellular ROS burden, omega-3 PUFAs limit the oxidative conditions that accelerate the Maillard reaction and promote the formation of AGEs from reducing sugars and macromolecules.

Furthermore, DHA and EPA inhibit the formation of methylglyoxal (MGO), a highly reactive dicarbonyl compound and potent glycating agent formed as a by-product of glycolysis, through reduction of hyperglycemia and oxidative stress. MGO drives the Maillard reaction approximately 250-fold more rapidly than glucose, making its suppression a critical target for limiting AGE accumulation (Rabbani and Thornalley, 2012). EPA and DHA supplementation has been shown to reduce fasting glucose and HbA1c in insulin-resistant and diabetic populations, thereby lowering the substrate availability for endogenous AGE synthesis (Mirhashemi et al., 2016; Kurt et al., 2016).

An emerging mechanistic pathway linking omega-3 fatty acids to reduced AGE-related inflammation operates through modulation of the gut microbiota. Aging is accompanied by gut dysbiosis, characterised by a relative reduction in anti-inflammatory taxa such as *Lactobacillus* and *Bifidobacterium* and an expansion of Gram-negative, lipopolysaccharide (LPS)-producing bacteria. The resulting increase in systemic endotoxemia activates Toll-like receptor 4 (TLR4)–NF-κB signalling, amplifying pro-inflammatory cytokine production, oxidative stress, and downstream AGE formation. Omega-3 fatty acids have been shown to modulate gut microbiota composition favourably, increasing the abundance of short-chain fatty acid-producing anti-inflammatory taxa and reducing LPS-producing Gram-negative bacteria, thereby attenuating systemic endotoxemia and its downstream inflammatory consequences (Costantini et al., 2017; Watson et al., 2018). This gut–inflammation–AGE axis represents an additional mechanistic route through which omega-3 supplementation may reduce glyco-oxidative burden, particularly in elderly populations where gut dysbiosis is prevalent and contributes to inflammaging.

## 6 OMEGA-3 fatty acids and advanced glycation end products: Evidence from the literature

### 6.1 *In vitro* and animal studies

Preclinical evidence supports a mechanistic relationship between omega-3 PUFA supplementation and reduced AGE formation and signalling. In experimental rodent models, n-3 PUFAs have been shown to attenuate the RAGE/oxidative stress/TNF-α axis, reducing renal AGE accumulation and improving markers of renal function in both diabetic and high-AGE-diet-fed animals (de Assis et al., 2013). In a study specifically designed to evaluate the extrinsic (dietary AGE) and intrinsic (diabetes-induced) pathways, n-3 PUFA supplementation significantly mitigated AGE-driven elevations in TNF-α, thiobarbituric acid reactive substances (TBARS), and RAGE protein expression in kidney tissue, demonstrating nephroprotective effects via antioxidant and anti-inflammatory pathways (de Assis et al., 2013).

*In vitro* studies have demonstrated that DHA inhibits RAGE expression in endothelial cells exposed to AGEs, attenuating RAGE-mediated adhesion molecule upregulation and oxidative stress responses. The activation of PPARγ by DHA has been specifically shown to downregulate RAGE transcription and attenuate AGE-induced proliferation of vascular smooth muscle cells (Wang et al., 2006). These findings identify RAGE suppression as a direct molecular target for omega-3 fatty acids, independent of their effects on AGE formation per se. Representative preclinical and *in vitro* studies are summarised in Table 1.

**TABLE 1:** Preclinical and *in vitro* evidence: omega-3 PUFAs and AGE/RAGE signalling.

Author (Year)	Study type	Model/Population	Intervention	Key findings	Relevance to Omega-3/AGE axis
de Assis et al. (2013)	*In vivo* (rodent)	Diabetic and high-AGE-diet-fed rats (dual-pathway model: Extrinsic dietary AGE and intrinsic diabetes-induced AGE)	n-3 PUFA supplementation (fish oil) versus control diet	• Reduced renal AGE accumulation • Reduced TNF-α • Reduced TBARS (lipid peroxidation) • Reduced RAGE protein expression in kidney • Improved renal function markers • Effects seen via both extrinsic and intrinsic AGE pathways	Demonstrates n-3 PUFAs attenuate the RAGE/oxidative stress/TNF-α axis *in vivo*, conferring nephroprotection via antioxidant and anti-inflammatory mechanisms
Wang et al. (2006)	*In vitro* (cell culture)	AGE-exposed VSMCs and endothelial cells	DHA treatment; PPARγ activation assessed	• Reduced RAGE transcription (PPARγ-mediated) • Reduced AGE-induced VSMC proliferation • Reduced adhesion molecule upregulation • Reduced oxidative stress in endothelial cells • PPARgamma identified as mechanistic mediator	Identifies RAGE suppression as a direct molecular target of DHA, independent of AGE formation. PPARγ activation links omega-3 intake to RAGE transcriptional downregulation

Abbreviations: AGE, advanced glycation end-product; DHA, docosahexaenoic acid; n-3 PUFA, omega-3 polyunsaturated fatty acid; RAGE, receptor for AGE; TBARS, thiobarbituric acid reactive substances; TNF-a, tumour necrosis factor-alpha; VSMC, vascular smooth muscle cell; PPARgamma, peroxisome proliferator-activated receptor gamma.

### 6.2 Clinical evidence: Omega-3 supplementation and circulating AGEs

Clinical evidence for the direct effect of omega-3 supplementation on AGE levels is emerging, though literature remains limited, particularly in elderly populations. A randomized, double-blind, placebo-controlled trial by Mirhashemi et al. (2016) in 60 patients with diabetic nephropathy demonstrated that supplementation with 1,000 mg/day of omega-3 fatty acids from flaxseed oil for 12 weeks resulted in a significant reduction in serum AGEs compared to placebo (2.3 AU versus +0.2 AU, p = 0.001) and a significant reduction in serum RAGE levels (p = 0.02). These findings were accompanied by significant improvements in inflammatory and cardiometabolic biomarkers.

These findings are informative regarding the potential of omega-3 supplementation to reduce circulating AGE-related markers; however, it should be noted that this study was conducted in patients with diabetic nephropathy, and the results cannot be directly extrapolated to aging populations without metabolic disease.

A subsequent study examined the effects of EPA and DHA supplementation (1.2 g/day) for 8 weeks on AGE subtypes in 38 patients with type 2 diabetes mellitus (T2DM). Significant reductions in fasting glucose, HbA1c, and pentosidine, a fluorescent AGE and established marker of long-term glyco-oxidative stress, were observed, while CML levels showed a trend toward reduction (Kurt et al., 2016). The reduction in pentosidine is particularly notable given its structural role in collagen cross-linking and its direct contribution to skin autofluorescence readings.

A systematic review by Mendes et al. (2022) synthesized six clinical trials evaluating the effects of dietary fat quality on AGE markers. The authors concluded that supplementation with omega-3 PUFAs consistently resulted in decreased concentrations of fluorescent AGEs and reduced RAGE expression, while increasing the expression of the protective AGE receptor 1 (AGER1), a scavenger receptor that promotes AGE clearance. Concurrently, Mediterranean diet interventions rich in MUFAs and omega-3s reduced serum AGE concentrations compared to Western diets high in saturated fats and dietary AGEs. It should be noted, however, that the AGE-lowering effects observed in Mediterranean diet interventions reflect the combined influence of multiple dietary components simultaneously—including reduced high-temperature food processing, higher antioxidant and polyphenol content, and altered macronutrient composition, and cannot be attributed specifically to omega-3 PUFAs. The clinical studies are summarised in Table 2.

**TABLE 2:** Emerging clinical evidence: omega-3 supplementation and circulating AGEs.

Author (Year)	Study design	Population	Intervention	Key findings	Relevance/Limitations
Mirhashemi et al. (2016)	RCT (double-blind, placebo-controlled)	n = 60 patients with diabetic nephropathy Omega-3 group (n = 30), mean age:63.5 ± 10.1 Placebo group (n = 30), mean age:63.1 ± 8.7	Omega-3 fatty acids from flaxseed oil, 1,000 mg/day for 12 weeks versus placebo	• Reduced serum AGEs: 2.3 AU versus +0.2 AU (placebo), p = 0.001 • Reduced serum RAGE levels (p = 0.02) • Significant improvements in inflammatory biomarkers • Significant improvements in cardiometabolic biomarkers	Provides early RCT evidence of reductions in both circulating AGEs and RAGE. Flaxseed oil (ALA-rich) limits generalisability to EPA/DHA-specific mechanisms
Kurt et al. (2016)	Clinical trial	Patients (n = 38) with T2DM Age: 45–75 years	EPA + DHA supplementation, 1.2 g/day for 8 weeks	• Reduced fasting glucose • Reduced HbA1c • Reduced pentosidine (fluorescent AGE; glyco-oxidative stress marker, p < 0.05) • CML: Trend toward reduction (non-significant)	Reduction in pentosidine is clinically significant given its role in collagen cross-linking and direct contribution to skin autofluorescence (SAF) readings

AGE, advanced glycation end-product; AGER1, AGE, receptor 1; ALA, alpha-linolenic acid; AU, arbitrary units; CML, N-(carboxymethyl)lysine; DHA, docosahexaenoic acid; EPA, eicosapentaenoic acid; HbA1c, glycated haemoglobin; PUFA, polyunsaturated fatty acid; RAGE, receptor for AGE; RCT, randomised controlled trial; SAF, skin autofluorescence; T2DM, type 2 diabetes mellitus.

### 6.3 Mechanistic pathways linking Omega-3s to AGE reduction

Integrating the preclinical and clinical evidence, at least four mechanistic pathways represent plausible links between omega-3 fatty acid supplementation and reduced AGE accumulation: (1) reduction of substrate availability for AGE formation through improved glycemic control and lower fasting glucose and HbA1c; (2) reduction of the oxidative micro-environment that accelerates AGE formation, through Nrf2-mediated antioxidant enzyme upregulation; (3) suppression of RAGE expression and AGE-RAGE signalling through PPARγ activation and NF-κB inhibition, limiting the perpetuation of the AGE-inflammation cycle; and (4) reduction of dietary AGE content indirectly by displacing pro-AGE saturated and trans fats in cell membrane phospholipids, altering the substrate pool for glycation reactions (Mendes et al., 2022; Mirhashemi et al., 2016; Wang et al., 2006). A fifth pathway, modulation of gut microbiota composition and attenuation of LPS-driven TLR4–NF-κB signalling (as described in Section 5.3), represents an additional mechanistically plausible route (Costantini et al., 2017; Watson et al., 2018).

**FIGURE 1:**
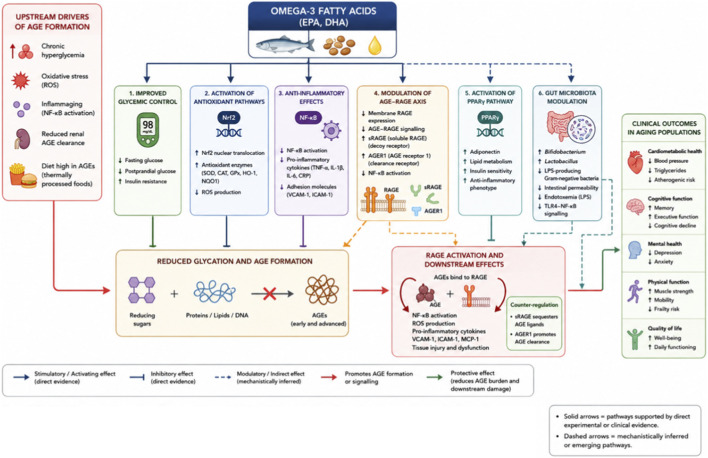
Mechanistic pathways through which omega-3 fatty acids (EPA and DHA) may reduce advanced glycation end product (AGE) formation, accumulation, and signalling during aging. Omega-3 fatty acids exert multiple complementary actions, including improved glycaemic control, suppression of NF-κB-mediated inflammatory signalling, activation of antioxidant defence pathways through Nrf2, modulation of the AGE–RAGE axis via reduced RAGE expression and increased activity of the protective receptors soluble RAGE (sRAGE) and AGE receptor 1 (AGER1), activation of PPARγ signalling, and potential regulation of gut microbiota composition and endotoxemia. These mechanisms collectively reduce oxidative stress, glyco-oxidative burden, and AGE–RAGE amplification, thereby attenuating chronic inflammation and tissue damage associated with aging. The downstream effects may contribute to improved cardiometabolic health, cognitive function, mental health, physical function, and overall quality of life in older adults. Solid arrows indicate pathways supported by direct experimental or clinical evidence, whereas dashed arrows represent mechanistically inferred, emerging, or indirect pathways.

The potential mechanisms linking omega-3 fatty acids to reduced AGE accumulation are summarized in Figure 1.

## 7 Effects of OMEGA-3 supplementation on secondary health outcomes in the elderly

### 7.1 Inflammatory markers

The evidence for omega-3 supplementation reducing systemic inflammatory markers in the elderly is substantial and consistent. A comprehensive systematic review and meta-analysis by Calder et al. (2020), synthesizing 45 RCTs, reported significant reductions in circulating CRP, IL-6, and TNF-α following omega-3 supplementation, with effect sizes that were larger in populations with elevated baseline inflammatory markers, a profile characteristic of elderly long-term care residents. Reductions in IL-6 are particularly clinically significant, as IL-6 is a primary driver of the acute-phase response, a mediator of muscle catabolism and sarcopenia, and independently predictive of mortality in older adults (Ferrucci and Fabbri, 2018).

The anti-inflammatory effects of omega-3 PUFAs in the elderly appear to operate both through classical eicosanoid pathway modulation and through the generation of SPMs, including resolvins and protectins. In elderly populations with a high burden of unresolved chronic inflammation, the capacity to generate SPMs, which declines with age, may be partially restored by omega-3 supplementation, shifting the balance from a pro-inflammatory to a pro-resolving phenotype (Serhan, 2017). This is mechanistically relevant to AGE reduction because the AGE-RAGE signalling axis is a potent driver of NF-κB-mediated inflammatory cytokine production; thus, reductions in upstream inflammation directly attenuate AGE-driven tissue damage.

### 7.2 Cardiometabolic health

Omega-3 fatty acids exert broad and well-characterized effects on cardiometabolic risk factors. Their most robustly demonstrated effect is triglyceride (TG) reduction: high-dose omega-3 supplementation (3–4 g/day EPA + DHA) consistently reduces fasting TG levels by 20%–50% in individuals with hypertriglyceridemia, an effect that is dose-dependent and mediated through reduced hepatic VLDL production and enhanced TG clearance (Skulas-Ray et al., 2019). At lower supplementation doses typical of dietary interventions (1–2 g/day), TG reductions are more modest (10%–15%) but clinically meaningful, particularly for individuals at elevated cardiovascular risk.

The landmark meta-analysis by Hu et al. (2019), encompassing 13 RCTs and 127,477 participants, demonstrated that omega-3 supplementation significantly reduced cardiovascular mortality (risk ratio 0.93, 95% CI 0.88–0.97) and improved major adverse cardiovascular outcomes, with EPA monotherapy showing the most pronounced effects. A subsequent meta-regression analysis further clarified that these cardiovascular benefits were most pronounced in individuals with established cardiovascular disease or high baseline cardiovascular risk (Mattumpuram et al., 2025), a profile highly prevalent in elderly long-term care residents.

Beyond lipid effects and cardiovascular events, omega-3 supplementation has demonstrated beneficial effects on blood pressure, particularly diastolic pressure in hypertensive individuals, endothelial function, arterial stiffness, insulin sensitivity, and fasting blood glucose (Mozaffarian and Wu, 2011). The 2022 AHA/ACC/HFSA guidelines now recommend omega-3 supplementation in patients with heart failure (Class IIb, Level B) to reduce cardiovascular hospitalization and mortality, representing the first incorporation of omega-3s into heart failure management guidelines (Heidenreich et al., 2022).

These cardiometabolic benefits are directly relevant to the AGE story: improvements in glycemic control reduce the substrate for AGE formation, while reductions in lipid peroxidation products and VLDL oxidation limit the formation of lipid-derived AGEs. The co-improvement of both AGE-forming conditions and cardiovascular risk factors by omega-3 supplementation therefore represents a biologically coherent and clinically synergistic therapeutic strategy for the elderly.

### 7.3 Cognitive function and neuroprotection

The brain is uniquely vulnerable to omega-3 deficiency. DHA constitutes approximately 40% of total PUFAs in brain gray matter, and is essential for synaptic membrane fluidity, neurotransmitter receptor function, and neuronal signalling (Calder, 2017). Aging is associated with progressive decline in brain DHA content, coinciding with structural and functional changes including hippocampal atrophy, reduced synaptic density, and impaired memory consolidation.

Clinical evidence for omega-3 supplementation and cognitive outcomes in elderly populations is accumulating but heterogeneous. A 2025 overview of systematic reviews, incorporating nine systematic reviews and 14 RCTs comprising 26,881 participants aged 40 years or older, reported a statistically significant but modest improvement in MMSE scores following omega-3 supplementation (pooled effect size 0.16, 95% CI 0.01 to 0.32, I^2^ = 42.8%) (Barros et al., 2025). This pooled estimate should be interpreted with caution: the MMSE is a global screening instrument with limited sensitivity to domain-specific cognitive changes, the moderate heterogeneity (I^2^ = 42.8%) reflects substantial variation across studies differing in population, formulation, and cognitive state, and pooling across these sources limits the interpretability of a single summary estimate. Critically, the benefits were most consistently observed in individuals with mild cognitive impairment (MCI), with 66.7% of MCI-specific RCTs reporting positive cognitive outcomes, compared to mixed results in cognitively normal older adults (Barros et al., 2025; Canhada et al., 2018).

A dose-response meta-analysis of 58 RCTs demonstrated that each 2,000 mg/day increment in omega-3 supplementation was associated with significant improvements in attention (SMD 0.98), language (SMD 0.98), and primary memory (SMD 0.87), with supplementation duration over 26 weeks associated with greater attentional gains. Among these cognitive domains, episodic memory, working memory, and attention appear most consistently responsive to omega-3 supplementation, whereas language and processing speed show less uniform responses across studies. The neuropsychological instruments used across trials are heterogeneous, encompassing MMSE, MoCA, digit span tasks, and various computerised batteries, which limits direct comparability of findings. A 2024 JAMA Network Open RCT demonstrated that high-dose omega-3 supplementation maintained neuronal integrity on MRI and slowed white matter lesion progression in older adults at high risk (Shinto et al., 2024).

The neuroprotective mechanisms of omega-3s in the aging brain are multifactorial and include: neuroinflammation suppression via SPM generation and NF-κB inhibition; reduction of amyloid-beta peptide production and aggregation; mitigation of tau hyperphosphorylation through GSK-3β inhibition; promotion of BDNF (brain-derived neurotrophic factor) expression supporting synaptic plasticity and neurogenesis; and attenuation of AGE-RAGE-driven neuroinflammation (Li et al., 2012; Mooldijk et al., 2024). Given the demonstrated link between elevated skin AGEs, smaller hippocampal volumes, and dementia risk in the Rotterdam Study, interventions that simultaneously reduce AGE burden and support brain DHA content offer a biologically plausible dual mechanism for cognitive preservation in elderly long-term care residents.

### 7.4 Mental health and neuroinflammatory pathways

The relationship between omega-3 fatty acids and mental health outcomes in aging can be conceptualised around three interconnected components: (1) AGE-RAGE-driven neuroinflammation as a mechanism contributing to psychiatric vulnerability; (2) omega-3 modulation of this pathway; and (3) clinical evidence for omega-3 effects on mental health outcomes.

Chronic low-grade inflammation has been consistently implicated in the pathophysiology of depression, with elevated levels of pro-inflammatory cytokines such as interleukin-6 (IL-6) and tumor necrosis factor-alpha (TNF-α) observed in affected individuals (Hueston and Deak, 2014; Osimo et al., 2020; Miller and Raison, 2016). Experimental studies further support this association, demonstrating that administration of inflammatory cytokines or inflammation-inducing agents can elicit depressive symptoms in otherwise healthy individuals, while anti-inflammatory interventions have been associated with improvements in depressive symptomatology (Reichenberg et al., 2001; Capuron et al., 2000; Köhler et al., 2014; Abbott et al., 2015).

In parallel, the accumulation of advanced glycation end products (AGEs) has been associated with psychiatric and cognitive outcomes, although findings remain heterogeneous. Some studies report no significant differences in AGE levels between individuals with neuropsychiatric disorders and healthy controls after adjustment for confounding factors (Yamashita et al., 2020), while others suggest that AGE levels may be influenced by clinical and metabolic characteristics, including pharmacological treatment (Hammoudeh et al., 2017). Longitudinal evidence further suggests that elevated AGE levels may precede and predict the persistence of psychiatric symptoms, including psychotic experiences (Hagen et al., 2020; Miyashita et al., 2021). The AGE-RAGE axis may contribute to this neuropsychiatric vulnerability through sustained NF-κB-mediated neuroinflammation in limbic and prefrontal regions, a process that omega-3 fatty acids may attenuate through NF-κB inhibition, Nrf2 activation, and SPM generation in neural tissue.

Omega-3 fatty acids have been investigated as adjunctive interventions in the management of depression, with evidence suggesting modest but heterogeneous benefits, particularly for EPA-rich formulations (Hoepner et al., 2021; Sikka et al., 2021; Mehdi et al., 2023). Mechanistically, omega-3 PUFAs may influence neurotransmitter systems involved in mood regulation, including serotonergic and dopaminergic pathways, partly through effects on neuronal membrane fluidity and receptor function. DHA depletion has been associated with impaired synaptic function, whereas EPA has been more consistently linked to improvements in depressive symptoms (McNamara et al., 2007; Mehdi et al., 2023).

Collectively, these findings suggest that inflammatory and oxidative pathways, including the AGE-RAGE neuroinflammatory axismay contribute to mental health outcomes, and that omega-3 supplementation may modulate these processes, although causal relationships remain to be fully established. However, the overall evidence remains heterogeneous and dependent on study design, population characteristics, and methods of AGE assessment.

### 7.5 Functional capacity and quality of life

Physical functional capacity, encompassing muscle strength, mobility, balance, and independence in activities of daily living, declines progressively with aging and is a major determinant of quality of life and institutionalization outcomes in the elderly (Cruz-Jentoft et al., 2019). Sarcopenia, the age-related loss of skeletal muscle mass and function, is highly prevalent in long-term care settings, affecting up to 33% of nursing home residents (Landi et al., 2012). The pathophysiology of sarcopenia involves chronic inflammation, oxidative stress, impaired protein synthesis, mitochondrial dysfunction, and motor neuron loss, all of which are potential targets of omega-3 supplementation.

Clinical evidence suggests that omega-3 supplementation can attenuate sarcopenia progression. A systematic review by Smith et al. (2015), conducted in healthy older adults, demonstrated that omega-3 PUFA supplementation significantly increased muscle protein synthesis rates and attenuated the anabolic resistance of aging skeletal muscle, an effect mediated through mTOR pathway activation and anti-inflammatory cytokine reduction. In elderly subjects specifically, supplementation with 3 g/day of omega-3 for 6 months increased muscle mass and improved both strength and functional performance measures, with effects likely mediated through reductions in IL-6-driven catabolism and enhanced anabolic signalling (Smith et al., 2015). Grip strength and gait speed, both validated predictors of mortality and disability in older adults, have shown improvements in RCTs of omega-3 supplementation in the elderly.

It is important to note that the available literature suggests omega-3 supplementation may improve muscle quality and strength more consistently than total lean mass. At least three complementary mechanisms may account for this pattern. First, reduction of AGE-mediated collagen cross-linking in the extracellular matrix of muscle tissue may improve fibre mechanics and tissue compliance, allowing for greater force transmission independent of changes in fibre volume. Second, attenuation of chronic IL-6- and TNF-α-driven catabolism reduces the catabolic cytokine burden on muscle protein turnover. Third, omega-3-associated improvements in neuronal membrane DHA content and function at the neuromuscular junction may enhance motor unit recruitment and strength output through improved synaptic transmission, independent of changes in muscle volume. These mechanisms may explain why grip strength and functional performance gains are more consistently reported than increases in total muscle mass across omega-3 supplementation trials in the elderly (Huang et al., 2020; Therdyothin et al., 2023; Tomczyk, 2024).

Health-related quality of life (HRQoL) assessment using validated multidimensional instruments such as the SF-36 provides a comprehensive perspective on the impact of interventions in elderly populations. Omega-3 supplementation has been associated with improvements in multiple SF-36 domains in older adults, including physical functioning, vitality, and mental health components, consistent with its broad anti-inflammatory, cardiometabolic, and neuroprotective effects (Calder et al., 2020). Psychological wellbeing, including depressive symptoms, also appears amenable to omega-3 supplementation: a meta-analysis by Sublette et al. (2011) demonstrated that EPA-predominant formulations were significantly effective in reducing depressive symptom scores, a finding of particular relevance in long-term care populations, where depression prevalence exceeds 40%.

These functional benefits may also be relevant to AGE biology, as chronic inflammation, oxidative stress, and collagen cross-linking contribute to impaired muscle quality, reduced tissue elasticity, and declining physical performance in older adults.

## 8 Discussion

The evidence synthesized in this review supports a biologically coherent and mechanistically grounded rationale for investigating omega-3 PUFA supplementation as a modulator of AGE accumulation in elderly populations. The convergence of aging-related oxidative stress, chronic inflammaging, impaired AGE clearance, and dietary patterns high in thermally processed foods creates a milieu uniquely conducive to accelerated AGE accumulation in elderly populations. Omega-3 fatty acids, through anti-inflammatory, antioxidant, glycemia-modulating, and RAGE-suppressive mechanisms, are mechanistically positioned to interrupt this cycle at multiple points.

The preclinical literature provides mechanistic proof-of-concept: n-3 PUFAs attenuate the RAGE/oxidative stress/TNF-α axis in rodent models, and DHA directly suppresses RAGE transcription via PPARγ activation *in vitro*. Emerging clinical evidence from trials in diabetic populations corroborates this, demonstrating significant reductions in circulating AGEs, RAGE, and pentosidine following omega-3 supplementation. However, this clinical evidence derives predominantly from studies in diabetic nephropathy and type 2 diabetes populations and cannot be directly extrapolated to elderly populations. No randomized controlled trial has yet evaluated omega-3 supplementation specifically targeting tissue AGE accumulation, measured by SAF or skin biopsy, as a primary endpoint in elderly cohorts. This gap in the evidence base is the most significant limitation of the current literature.

Safety and tolerability are essential translational considerations for omega-3 supplementation in elderly populations, who are typically polymedicated and have impaired physiological reserve. Gastrointestinal adverse effects including nausea, eructation, and loose stools—are the most commonly reported side effects and may affect adherence, particularly in elderly individuals with pre-existing gastrointestinal conditions or swallowing difficulties. Fishy aftertaste from standard fish oil formulations may further limit compliance, and enteric-coated or algal-derived preparations may improve acceptability in this group.

Omega-3 supplementation at doses of 3 g/day or higher has been associated with a modest increase in bleeding time, and caution is warranted in individuals receiving anticoagulant therapy (e.g., warfarin, direct oral anticoagulants) or antiplatelet agents. While clinically significant haemorrhagic events are rare at typical supplementation doses, regular INR monitoring and clinical review are prudent in polymedicated long-term care residents. Additionally, certain ethyl ester formulations of omega-3 PUFAs have been associated with modest increases in LDL-cholesterol at high doses, which should be considered in individuals with established cardiovascular disease or those receiving lipid-lowering therapy.

Practical implementation factors relevant to older populations include pill burden, storage conditions to minimise oxidation of omega-3 preparations, and the availability of alternative formulations for individuals with dysphagia or swallowing difficulties. These aspects should be considered when designing future clinical trials and translating omega-3 supplementation strategies into routine care for older adults.

The existing clinical evidence is limited by several important considerations: studies have been predominantly conducted in younger diabetic populations rather than elderly individuals; dosing regimens have been heterogeneous and not always focused on EPA/DHA specifically; and most trials have relied on circulating rather than tissue-bound AGE markers, which may underestimate the glycation burden in long-lived structural proteins such as collagen.

To date, randomized controlled trial data evaluating the effects of omega-3 supplementation on tissue AGE accumulation using non-invasive measures such as skin autofluorescence remain limited, particularly in elderly long-term care populations. This gap is clinically significant given the strong predictive validity of SAF for cardiovascular and all-cause mortality, cognitive decline, and functional outcomes, precisely the health domains most relevant to the older adults. Future research should prioritize adequately powered, placebo-controlled RCTs in this population, using SAF as a primary endpoint, with pre-specified secondary outcomes encompassing cardiometabolic, cognitive, functional, and quality-of-life measures.

## 9 Conclusion

The evidence synthesized in this narrative review supports a biologically plausible and mechanistically grounded relationship between omega-3 polyunsaturated fatty acid supplementation and modulation of advanced glycation end product (AGE) accumulation in the context of aging. The aging process is associated with a pro-oxidative and pro-inflammatory environment that promotes glyco-oxidative stress and tissue AGE accumulation, particularly in elderly individuals residing in long-term care facilities. Omega-3 fatty acids, through their anti-inflammatory, antioxidant, glycemia-modulating, and RAGE-suppressive properties, represent a mechanistically plausible and translational strategy for modulating AGE-related pathways. Preclinical evidence in this area is substantial and biologically coherent. Early clinical evidence from trials in diabetic and metabolically dysregulated populations is promising, demonstrating reductions in circulating AGE-related markers and downstream inflammatory and oxidative processes. However, direct evidence in elderly populations is currently absent, and the findings from diabetic cohorts cannot be assumed to translate directly to this population. However, the current clinical evidence base remains limited by population heterogeneity, variability in study design and dosing regimens, and a predominant reliance on circulating rather than tissue-based AGE measures. Skin autofluorescence, as a validated, non-invasive, and clinically predictive measure of cumulative AGE burden, represents a promising endpoint for future intervention studies. Well-designed randomized controlled trials in elderly populations are needed to determine whether omega-3 supplementation can meaningfully reduce tissue AGE accumulation and translate into improvements in clinically relevant outcomes, including functional capacity, cognitive health, and quality of life. Such studies would provide important insights into the potential role of targeted nutritional interventions in mitigating glyco-oxidative stress and promoting healthier aging trajectories.
